# Omega-3 fatty acids abrogates oxido-inflammatory and mitochondrial dysfunction-associated apoptotic responses in testis of tamoxifen-treated rats

**DOI:** 10.3389/fnut.2024.1443895

**Published:** 2024-08-01

**Authors:** Adeyemi Fatai Odetayo, Roland Eghoghosoa Akhigbe, Moses Agbomhere Hamed, Morufu Eyitayo Balogun, David Tolulope Oluwole, Luqman Aribidesi Olayaki

**Affiliations:** ^1^Department of Physiology, Faculty of Basic Medical Sciences, Federal University of Health Sciences, Ila Orangun, Nigeria; ^2^Reproductive Biology and Toxicology Research Laboratory, Oasis of Grace Hospital, Osogbo, Nigeria; ^3^Department of Physiology, Ladoke Akintola University of Technology, Ogbomoso, Nigeria; ^4^Department of Medical Laboratory Science, Afe Babalola University, Ado Ekiti, Nigeria; ^5^The Brainwill Laboratories and Biomedical Services, Osogbo, Nigeria; ^6^Department of Physiology, Crescent University, Abeokuta, Nigeria; ^7^Department of Physiology, University of Ilorin, Ilorin, Nigeria

**Keywords:** anticancer drugs, nuclear factor erythroid 2-related factor 2 (Nrf2) and nuclear factor-kappa B (NF-κB) signaling, selective estrogen receptor modulators, testicular function, cytochrome c

## Abstract

**Background:**

Tamoxifen (TAM) is a widely used drug in patients with gynecomastia and breast cancer. TAM exerts its anticancer effects via its antiestrogenic activities. Unfortunately, TAM has been reported to exert gonadotoxic effects on male testes. Therefore, this study was designed to explore the possible associated mechanisms involved in TAM-induced testicular dysfunction and the possible ameliorative effects of omega-3 fatty acids (O3FA).

**Methodology:**

Animals were randomly divided into control, O3FA, TAM, and TAM + O3FA. All treatment lasted for 28 days.

**Results:**

TAM exposure impaired sperm qualities (count, motility, and normal morphology) and decreased testicular 3β-HSD and 17β-HSD. It was accompanied by a decline in serum testosterone and an increase in estradiol, luteinizing and follicle-stimulating hormones. These observed alterations were associated with an increase in testicular injury markers, oxido-inflammatory response, and mitochondria-mediated apoptosis. These observed alterations were ameliorated by O3FA treatments.

**Conclusions:**

O3FA ameliorated TAM-induced testicular dysfunction in male Wistar rats by modulating XO/UA and Nrf2/NF-kb signaling and cytochrome c-mediated apoptosis in TAM-treated rats.

## Introduction

Tamoxifen (TAM; Z-1-[4-(2-dimethylaminoethoxy)-phenyl]-1,2-diphenyl-1-butene) is a synthetic nonsteroidal estrogen agonist-antagonist antineoplastic agent (1, 2). TAM is the major anti-estrogen therapy for the management of hormone receptor-positive breast cancer in pre-menopausal women (3). TAM has also been recommended for the management of gynecomastia in males (4). In fact, TAM is recommended for pubertal gynecomastia once it is accompanied by significant pain, irrespective of the disc size (5). TAM is believed to majorly act through its inhibitory effect on estradiol binding at the ligand-binding domain of the estrogen receptor (ER) alpha and blockage of estrogen receptor interaction with co-activator proteins (6, 7). However, TAM has also been shown to act as an estrogen agonist. These dual actions on estrogen could depend on the type of species, cell types, tissue, and organs (8, 9). In humans and rats, TAM primarily exhibits antiestrogenic activities with residual estrogenic effects (10). Apart from these estrogenic effects, TAM also acts via different signaling proteins such as protein kinase C, mitogen-activated protein kinases, and c-jun N-terminal kinase (JNK) and also distorts bcl-2-like protein 4 (BAX)/B-cell lymphoma 2 (BCL-2) ratio. Furthermore, TAM stimulates the mitochondrial permeability transition and cytochrome C release, which eventually results in increased apoptosis (11). With the increasing usage of TAM for the management of gynecomastia (4) and possibly benign prostatic hyperplasia (12), attention has been drawn to its possible testicular toxic effects.

TAM has been shown to impair spermatogenesis and steroidogenesis (13). TAM administration has also been shown to disrupt the hypothalamic-pituitary-gonadal (HPG)-axis (14) responsible for maintaining testicular functions. TAM-induced testicular toxicities could be associated with reactive oxygen species (ROS) generation (15), which are capable of reacting with the cellular DNA, proteins, and lipids to form DNA-adducts, protein crosslink, and lipid peroxidation products (16) in the testis. As a result, these activities can create oxidative stress (redox imbalance), inflammatory response, mitochondrial dysfunction, uncontrolled cell death, and impair testicular cells integrity and functionality. Hence, this study sought to establish a supplement for managing TAM-induced gonadotoxicity in patients who require TAM treatment.

Nutritional supplements can be recommended for the prevention and management of toxicants-induced health disorders. Omega-3 fatty acids (O3FA) is one of these natural supplements that has been shown to possess various pharmacological and biological activities (17, 18). O3FA are essential fatty acids commonly found in plants and marine life. They are referred to as essential fatty acids because they cannot be synthesized in the body; they can only be obtained from diets. O3FA are required for different functions such as growth, brain development, vision, and fertility enhancement (19). O3FA might be performing these functions via its anti-inflammatory (20), anti-oxidant (21, 22), and anti-apoptotic (23) activities.

Despite O3FA's established protective activities, no study has explored its possible ameliorative role on TAM-induced testicular injury. Hence, we hypothesize that O3FA might attenuate TAM-induced testicular toxicity in male Wistar rats. The findings from this study will establish O3FA as a supplement that can be introduced as an adjunct therapy together with TAM.

## Materials and methods

### Chemicals/reagents

TAM 20 mg was purchased from Milpharm, Ltd, UK, while O3FA was procured from Gujarat Liqui Pharmacaps Pvt. Ltd. Vadodara, Gujarat, India. Each of the O3FA capsules consists of eicosapentaenoic acid (EPA) and docosahexaenoic acid (DHA) in 3:2. All other chemicals used in this study except otherwise stated were of analytical grades and were procured from Sigma (MS, USA).

### Ethical consideration

The animals were humanly handled in accordance with the Guidelines for Laboratory Animal Care published by the National Institute of Health (NIH). The experimental protocol complied with the US NAS guidelines, and ethical approval was obtained from the Institutional Ethical Review Committee (UERC/ASN/2022/2396).

### Animals

Twenty-four (24) male Wistar rats (aged 10–12 weeks and weighing 180–200 g) were purchased from the Biochemistry Department, University of Ilorin, and housed in standard ventilated cages. The rats were allowed free access to feed and water under a normal 12-h light and darkness cycle.

### Experimental procedure

The animals were allowed to acclimatize for 2 weeks before they were randomly divided into 4 groups (*n* = 6 groups): Group 1: Control (Cntrl), vehicle-treated animals with 0.5 ml of corn oil, Group 2: animals treated with 300 mg/kg of O3FA, Group 4: animals exposed to 0.4 mg/kg of TAM, Group 5: animals co-treated with 0.4 mg/kg of TAM and 300 mg/kg of O3FA. All treatments were via oral gavage and lasted for 28 days. The dose of 0.4 mg/kg used in this study has been earlier reported as the most effective dosage of TAM for antifertility studies (7) and is similar to that of Motrich et al. (1) and Lee et al. (15), while the 300 mg/kg of O3FA was the most effective dosage based on the reports from our previous findings (19, 22).

The study was terminated 24 h after the last treatment, and animals were sacrificed via an intraperitoneal administration of ketamine (40 mg/kg) and xylazine (4 mg/kg) (24). Blood samples were obtained via cardiac puncture and put into plain bottles. The obtained blood samples were centrifuged at 3,000 rpm for 10 min, and the obtained serum was used for hormonal assay. Both testes were removed, and the surrounding tissues were separated. The left testes were homogenized in phosphate buffer for biochemical assays, while the right testes were preserved with bouin solution for histology.

### Sperm analysis

Caudal epididymis was meticulously cut into a clean petri dish, and sperm count, motility, and abnormal sperm morphology were estimated based on previous methods (25, 26). Briefly, for sperm motility, cauda epididymis was cut with surgical blade; the spermatozoa released onto a sterile glass slide and then diluted with pre-warmed 2.9% sodium citrate dehydrate solution. The glass slide was covered with a coverslip, and sperm motility was evaluated under microscope by examining at least ten microscopic fields at × 40 magnification. For sperm count, the cauda epididymis was gently crushed in normal saline and filtered with a nylon mesh to obtain the sperm suspension. Five μL of the sperm suspension was mixed with 95 μl of 0.35% formalin containing 0.25% trypan blue and 5% NaHCO3. A fraction (10 μl) of the diluted spermatozoa was placed on the haemocytometer, allowed to sediment for 5 min, and then counted using the Improved Neubauer chamber and a light microscope at × 40. For sperm morphology, The abnormalities in the head, middle-piece and tail (tailless head, bent mid piece, curved mid-piece, headless tail, bent tail, curved tail, looped tail) were counted and classified as documented by Bloom (27) and Parkinson (28).

### Steroidogenic enzymes

Testicular 3 beta-hydroxysteroid (3β-HSD) (29, 30) and 17 beta-hydroxysteroid (17 β-HSD) dehydrogenase (30, 31) were estimated as previously established respectively. “For 3β-HSD, testicular tissue was homogenized, and the supernatant was carefully separated. 1 ml of the supernatant was mixed with 1 ml of 100 μmol sodium pyrophosphate buffer (pH 8.9), 30 μg of dehydroepiandrosterone in 40 μl of ethanol, and 960 μl of 25% BSA. The mixture was then incubated and 0.5 μmol of NAD was added. The absorbance was read spectrophotometrically at a wavelength of 340 nm using a blank as reference. For testicular 17β-HSD, 1 ml of the supernatant obtained from the testicular sample was mixed with 1 ml of 440 μmol sodium pyrophosphate buffer (pH 10.2), 40 μl of ethanol containing 0.3 μmol of testosterone, and 960 μl of 25% BSA. The mixture was incubated and 1.1 μmol of NAD was added in a U 2,000 spectrophotometer cuvette at 340 nm against a blank.”

### Reproductive hormones

The serum levels of luteinizing hormone (LH), follicle-stimulating hormone (FSH), testosterone, and estradiol (Bio-Inteco, UK) were determined using an ELISA method according to the manufacturer's description.

### Testicular histology

Histology was performed according to the established method (32, 33). The preserved testis in bouin solution was dehydrated using ethanol series and cleared with toluene. The cleared testes were embedded and blocked in paraffin wax. After that, 5 μm thick paraffin sections were stained with hematoxylin and eosin (H&E). Testicular biopsy/Johnsen score was estimated as previously described (30, 34).

### Testicular injury markers

Testicular lactate dehydrogenase (LDH) and Gamma-glutamyl transferase (GGT) activities were determined as described by the manufacturer (Agape Diagnostics Ltd.). Additionally, testicular lactate concentration was evaluated based on the manufacturer's guideline (EnzyChrom, ELAC-100).

### Oxidative stress markers

Testicular malondialdehyde (MDA) level was assayed as previously reported (35, 36). In addition, testicular glutathione (GSH), glutathione peroxidase (GPx), Glutathione-S-transferase (GST), superoxide dismutase (SOD), and catalase (CAT) activities were assayed based on established methods (30, 37, 38).

“Malondialdehyde (MDA), a marker of oxidative stress, was determined as previously documented based on the generated amount of thiobarbituric acid reactive substance (TBARS) during lipid peroxidation. This method involves the reaction between 2- thiobarbituric acid (TBA) and malondialdehyde, a byproduct of lipid peroxidation, by analyzing the pink chromogen complex [(TBA) 2-malondialdehyde adduct] formed upon heating at acidic pH. The sample (200 μl) was first treated with 500 μl of Trichloroacetic acid (TCA) to remove proteins and centrifuged at 3,000 rpm for 10 min. Next, 1 ml of 0.75% TBA was added to 0.1 ml of the supernatant and heated in a water bath at 100°C for 20 min, then cooled with ice water. The absorbance of the sample/standard was then read at 532 nm using a spectrophotometer and compared to a blank. The concentration of TBARS was determined by extrapolating from a standard curve.

For GSH, an aliquot of the sample was deproteinized by adding an equal volume of 4% sulfosalicylic acid, and was centrifuged at 4,000 rpm for 5 min. 0.5 ml of the supernatant was then added to 4.5 ml of Ellman's reagent. A blank was prepared by mixing 0.5 ml of the diluted precipitating agent with 4.5 ml of Ellman's reagent. The level of GSH was calculated by measuring the absorbance at 412 nm.

For catalase, 1:29 dilution of the sample was made by mixing 1 ml of the supernatant of the testicular homogenate with 19 ml of diluted water. 4 ml of H2O2 solution (800 μmoles) and 5 ml of phosphate buffer were added to a 10 ml flat bottom flask. 1 ml of the diluted enzyme preparation was mixed into the reaction mixture by gentle swirling at 37°C. Samples of the reaction mixture were withdrawn at 60 s intervals, and the H_2_O_2_ content was determined by blowing 1 ml of the sample into 2 ml dichromate/acetic acid reagent. Catalase levels in the sample were determined by comparing the absorbance at 653 nm to that of a certified catalase standard.

For GPx, the sample was incubated at 37°C for 3 min, then 0.5 ml of 10% trichloroacetic acid (TCA) was added and the mixture was centrifuged at 3,000 rpm for 5 min. The supernatant was then mixed with 2 ml of phosphate buffer and 1 ml of 5′- 5′- dithiobis-2-dinitrobenzoic acid (DTNB) solution, and the absorbance was measured at 412 nm using a blank as reference. The GPx activity was determined by plotting a standard curve and determining the concentration of remaining GSH from the curve.

The activity of glutathione-S-transferase in testicles was also measured. This method utilizes the enzyme's high activity with 1-chloro-2,4-dinitrobenzene as a substrate. The assay was performed at 37°C for 60 s and the absorbance was read at 340 nm after comparing it with a blank sample.

For SOD, a 1:10 dilution of the sample was made using 1 ml of sample and 9 ml of distilled water. 0.2 ml of the diluted sample was added to 2.5 ml of 0.05 M carbonate buffer (pH 10.2) and the reaction was initiated by adding 0.3 ml of freshly prepared 0.3 mM adrenaline. The mixture was mixed and the increase in absorbance was monitored at 480 nm every 30 s for 150 s using a spectrophotometer. A reference cuvette containing 2.5 ml buffer, 0.3 ml substrate (adrenaline), and 0.2 ml water was also used.”

### Inflammatory markers

Testicular tumor necrotic factor-alpha (TNF-α) and interleukin 6 (IL-6) were assayed using ELISA kits (Solarbio, China). Also, testicular myeloperoxidase (MPO) was estimated according to previously reported methods (30, 39), while testicular nitric oxide (NO) concentration was determined based on the Griess reaction (40).

Briefly, “the method for measuring MPO is based on the ability of myeloperoxidase to catalyze the oxidation of guaiacol to oxidized guaiacol in the presence of hydrogen peroxide. The oxidized form of guaiacol has a brown color, which is measured spectrophotometrically at a wavelength of 470 nm. The intensity of the color produced is proportional to the concentration of oxidized guaiacol produced in the reaction, thus providing a measure of myeloperoxidase activity.

For NO, a mixture of 100 μl of Griess reagent, 300 μl of a nitrate-containing testicular homogenate, and 2.6 ml of deionized water were incubated for 30 min at room temperature in a spectrophotometer cuvette. A blank was prepared by mixing 100 μl of Griess reagent and 2.9 ml of deionized water. The absorbance of the nitrate-containing sample was measured at 548 nm in relation to the reference sample.”

### Xanthine oxidase/uric acid

The activities of testicular xanthine oxidase (XO) were determined based on the method of Zahide and Bahad (41), while a colorimetric method was used for uric acid (UA) concentration (Precision, UK).

### Transcriptional factors

Testicular nuclear factor kappa B (NFkB) and nuclear factor erythroid 2-related factor 2 (Nrf2) levels were determined using the ELISA method (Elabscience Biotechnology Inc., USA).

### Apoptotic markers

Testicular BCl-2, cytochrome C, and caspase 3 activities were determined by an ELISA method as described by the manufacturer (Elabscience Biotechnology Inc., USA). At the same time, the testicular DNA Fragmentation Index (DFI) was estimated according to the method of Perandones et al. (42). Five ml each of testicular homogenate supernatant and pellet were treated with 3 ml of freshly prepared diphenylamine (DPA) reagent for color development. The solution was incubated at 37°C for 16 to 24h. The absorbance of light green/yellowish-green supernatant was read spectrophotometrically at 620 nm. The percentage of fragmented DNA was calculated by dividing the absorbance of the homogenate supernatant by the sum of the absorbance of the pellet and the absorbance of the supernatant.

### Statistical analysis

Data were analyzed using a one-way analysis of variance (ANOVA) followed by Tukey's *post hoc* test using GraphPad PRISM 5 software, and they were reported as mean ± standard deviation. Also, all *P* values below 0.05 were classified as statistically significant.

## Results

**Figure 1:**
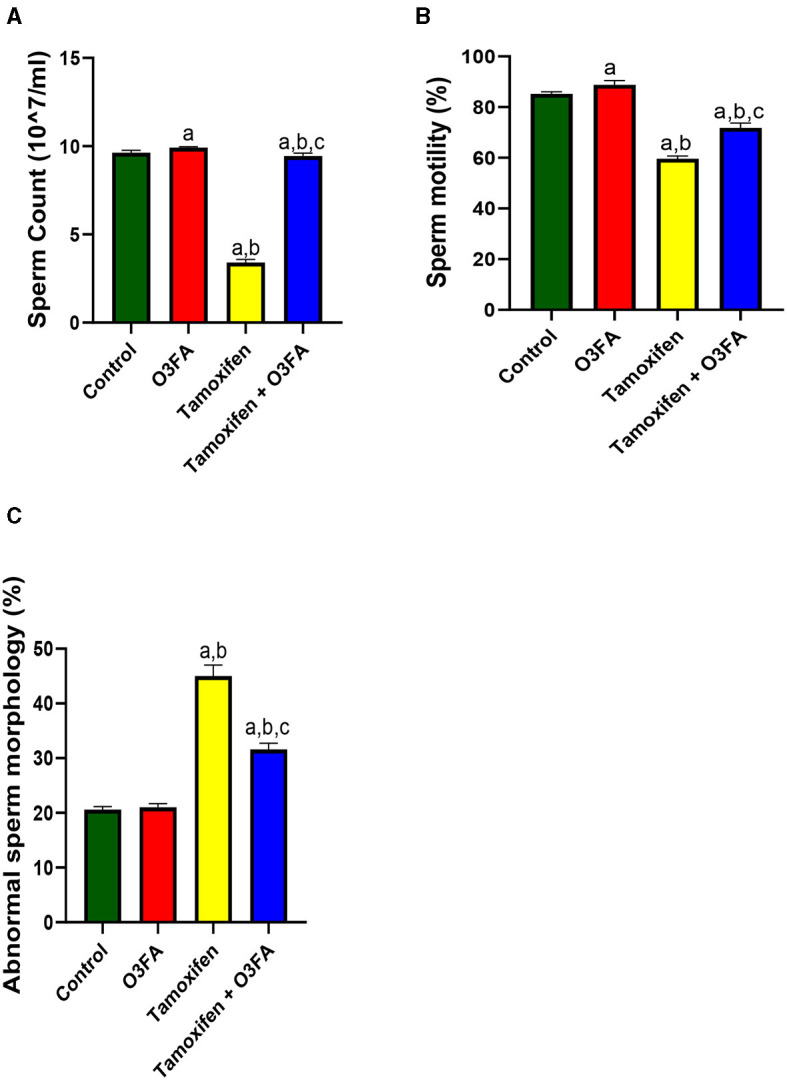
Effect of O3FA on sperm **(A)** count **(B)** motility **(C)** abnormal morphology in TAM exposed rats. ^a^*P* < 0.05 vs. control, ^b^*P* < 0.05 vs. O3FA; ^c^*P* < 0.05 vs. TAM. Data were analyzed by one way ANOVA and Tukey's *post-hoc* test. O3FA, Omega-3 fatty acids; TAM, Tamoxifen.

As shown in Figure 1, TAM exposure led to a significant decrease in sperm count (*p* < 0.0001), motility (*p* < 0.0001), and an increase in abnormal morphology (*p* < 0.0001) compared with the control and O3FA groups. These impaired sperm qualities were ameliorated by O3FA treatment.

**Figure 2:**
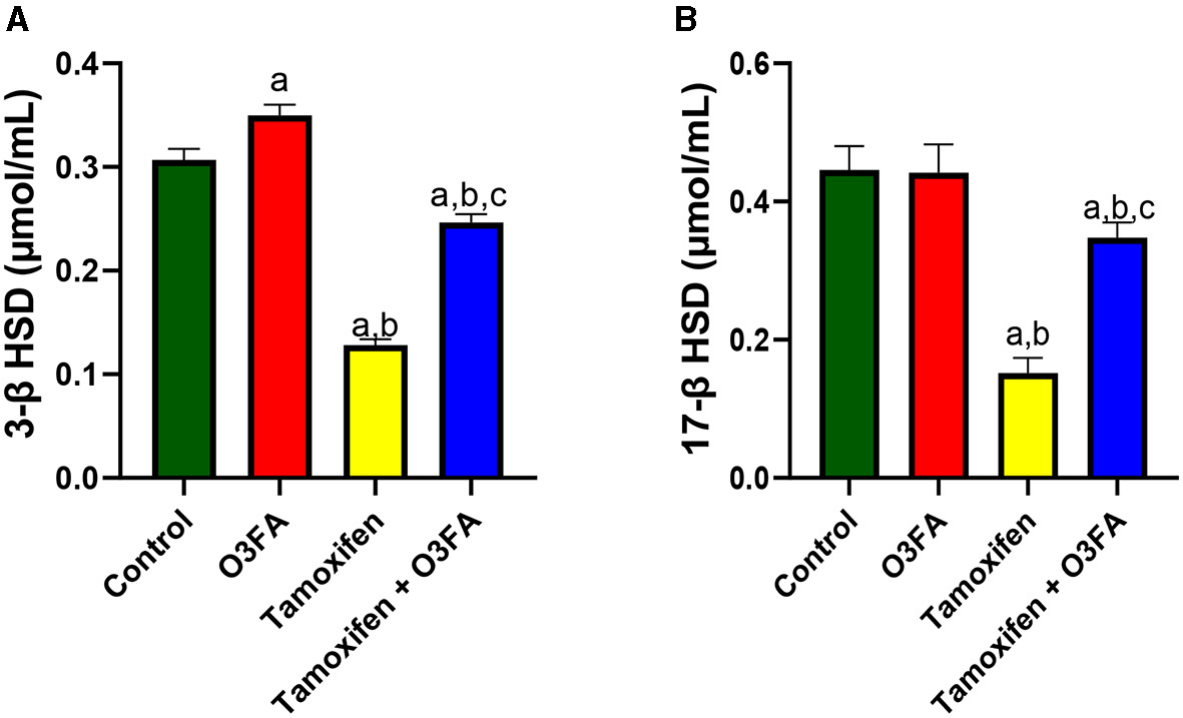
Effect of O3FA on testicular **(A)** 3β-HSD **(B)** 17β-HSD in TAM exposed rats. ^a^*P* < 0.05 vs. control, ^b^*P* < 0.05 vs. O3FA; ^c^*P* < 0.05 vs. TAM. Data were analyzed by one way ANOVA and Tukey's *post-hoc* test. O3FA, Omega-3 fatty acids; TAM Tamoxifen; 3β-HSD, 3 beta-hydroxysteroid dehydrogenase; 17β-HSD, 17 beta-hydroxysteroid dehydrogenase.

In the same vein, TAM administration impaired steroidogenic enzymatic activities, evidenced by a significant decrease in 3β-HSD (*p* < 0.0001) and 17β-HSD (*p* < 0.0001) compared with the control and O3FA groups (Figure 2). These observed decreases were abrogated in animals in the TAM+ O3FA group.

**Figure 3:**
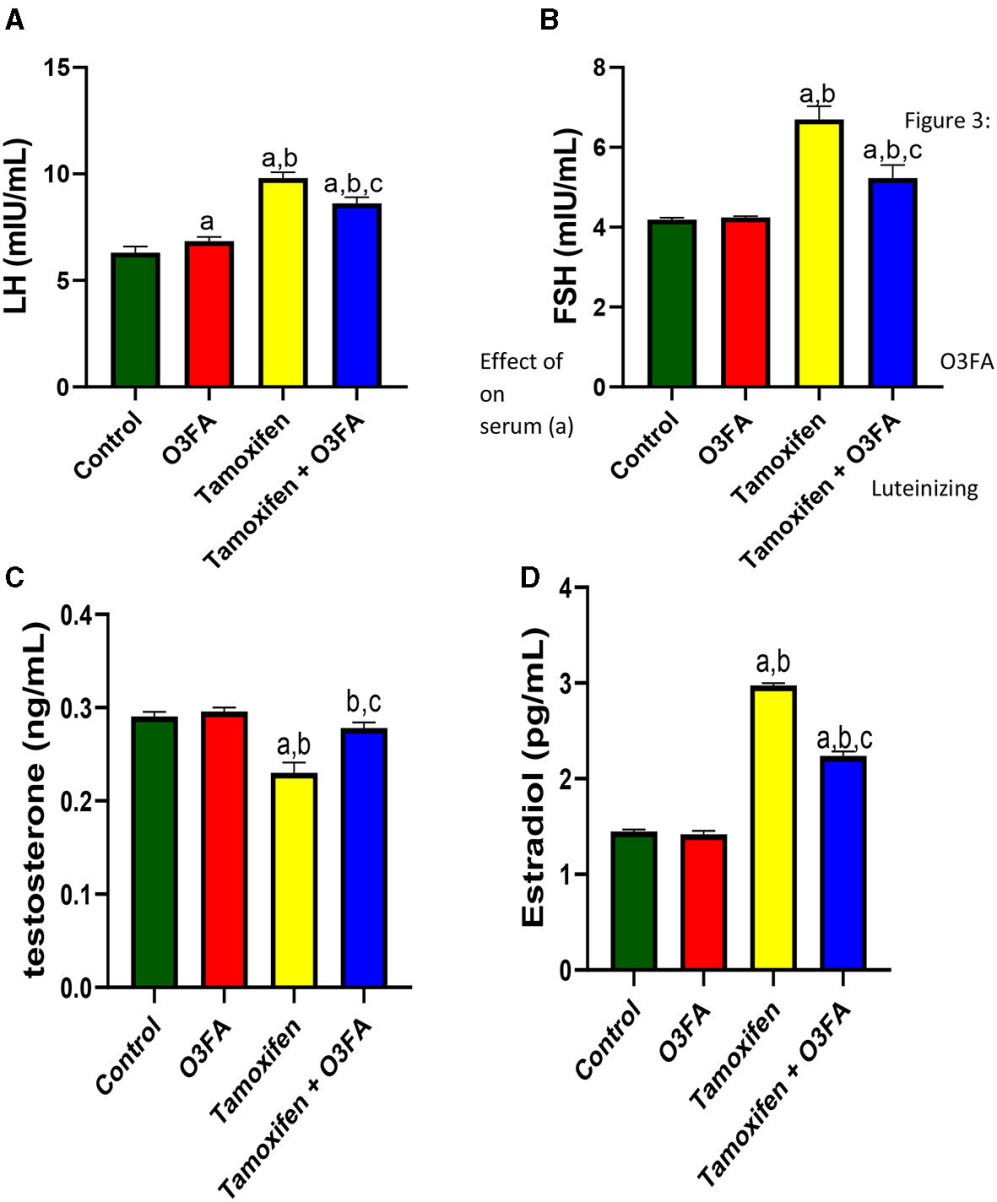
Effect of O3FA on serum **(A)** Luteinizing hormone (LH) **(B)** Follicle stimulating hormone (FSH) **(C)** testosterone **(D)** estradiol in TAM exposed rats. ^a^*P* < 0.05 vs. control, ^b^*P* < 0.05 vs. O3FA; ^c^*P* < 0.05 vs. TAM. Data were analyzed by one way ANOVA and Tukey's *post-hoc* test. O3FA, Omega-3 fatty acids; TAM, Tamoxifen.

Furthermore, compared with the control groups, animals administered with TAM had a significant increase in serum LH (*p* < 0.0001), FSH (*p* < 0.0001), and estradiol and a decrease in testosterone (Figure 3). TAM and O3FA co-administration blunted these observed hormonal imbalances.

**Figure 4:**
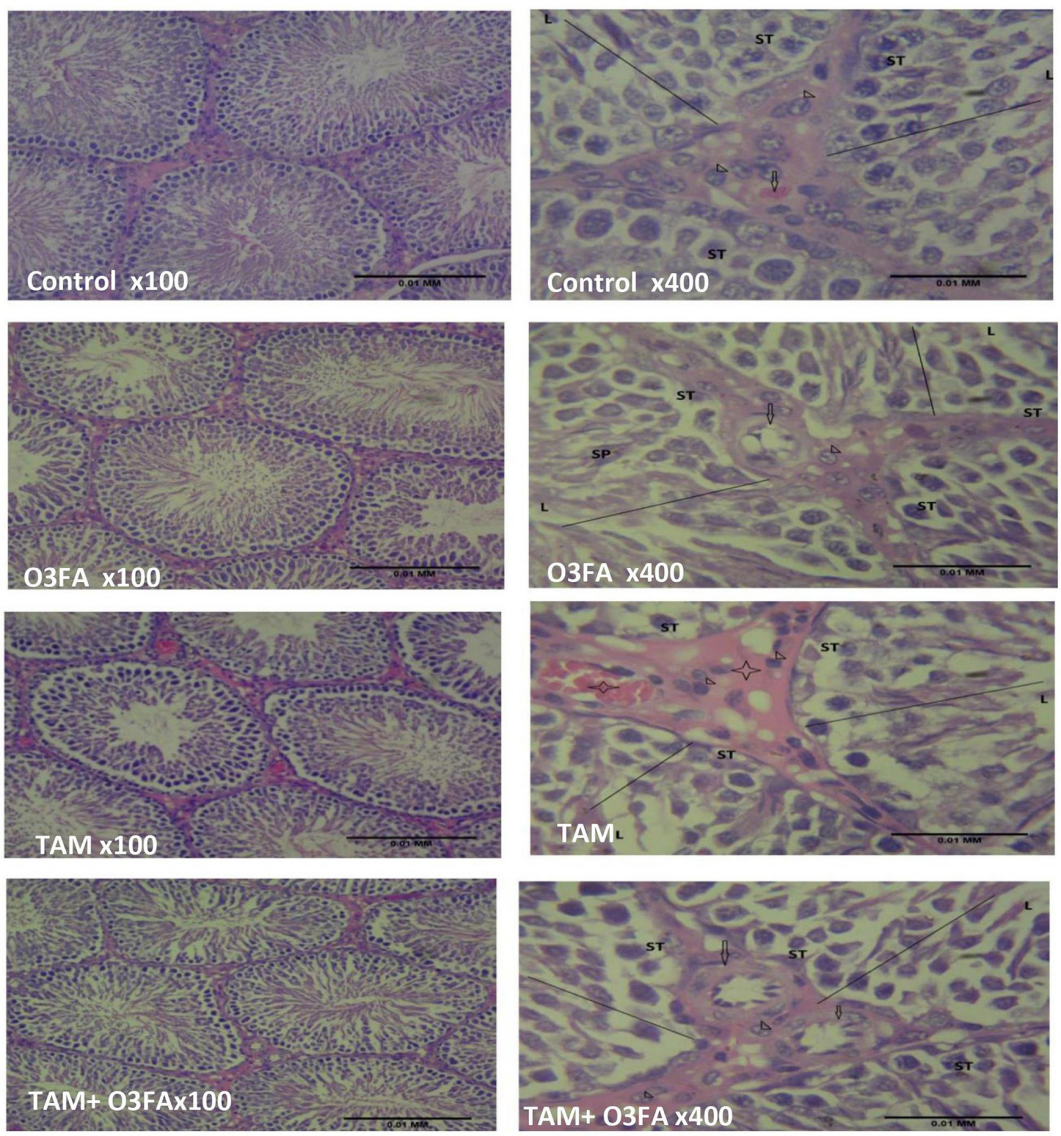
Testicular histology. Control, O3FA, and TAM + O3FA: section shows the testicular tissue composed of coils of seminiferous tubules (ST) with a defined lumen (L) containing sperm cells (SP), the seminiferous tubules contained germinal epithelium with germ cells at varying degree of maturation (line). The interstitium, contained blood vessel (arrow) which is free of collection and contained interstitial cells of Leydig (arrow head) appearing unremarkable. TAM, The blood vessels (black star) within the interstitium (plain star) appeared congested, and the interstitial cells of Leydig (arrow head) appears unremarkable.

**Figure 5:**
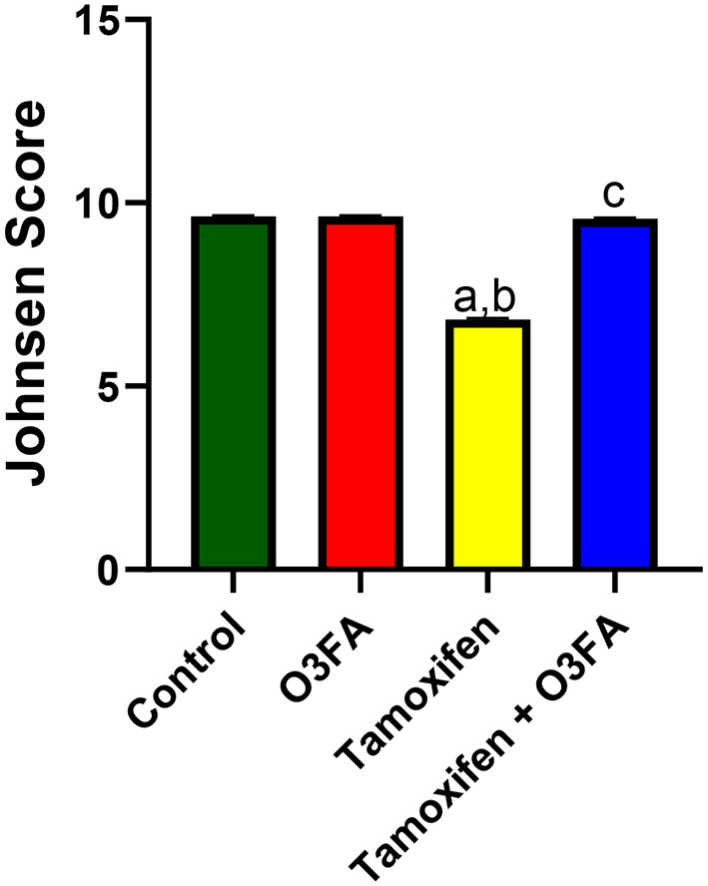
Effect of O3FA on Johnsen Score in TAM exposed rats. ^a^*P* < 0.05 vs. control, ^b^*P* < 0.05 vs. O3FA; ^c^*P* < 0.05 vs. TAM. Data were analyzed by one way ANOVA and Tukey's *post-hoc* test. O3FA, Omega 3 fatty acids; TAM, Tamoxifen.

Histopathological findings revealed features consistent with normal testicular tissue of animals in the control, O3FA, and TAM + O3FA groups, while their counterparts in the TAM group exhibited histological features that suggest cellular reaction to injury and inflammatory response (Figure 4). Also, a decrease in Johnsen score was observed in TAM-exposed animals compared with the controls (Figure 5). This alteration was ameliorated in animals that received TAM and O3FA co-treatment.

**Figure 6:**
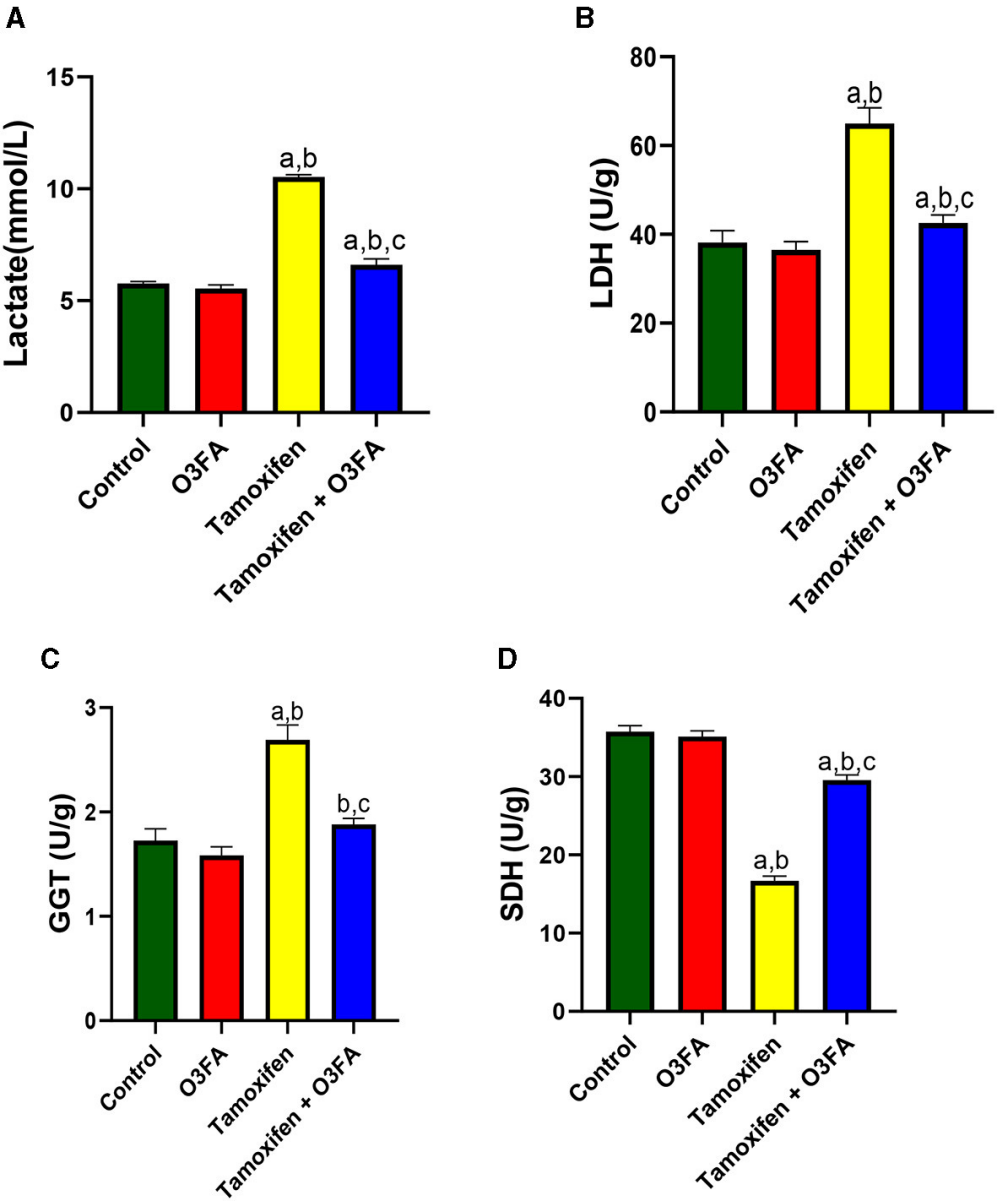
Effect of O3FA on testicular **(A)** lactate **(B)** lactate dehydrogenase (LDH) **(C)** Gamma-glutamyl transferase (GGT) **(D)** sorbitol dehydrogenase (SDH) in TAM exposed rats. ^a^*P* < 0.05 vs. control, ^b^*P* < 0.05 vs. O3FA; ^c^*P* < 0.05 vs. TAM. Data were analyzed by one way ANOVA and Tukey's *post-hoc* test. O3FA, Omega 3 fatty acids; TAM, Tamoxifen.

Additionally, TAM administration led to a significant increase in testicular lactate, LDH, and GGT and a decrease in testicular SDH (Figure 6) compared with the controls. These observed increases in testicular injury markers were ameliorated in animals treated with TAM and O3FA.

**Figure 7:**
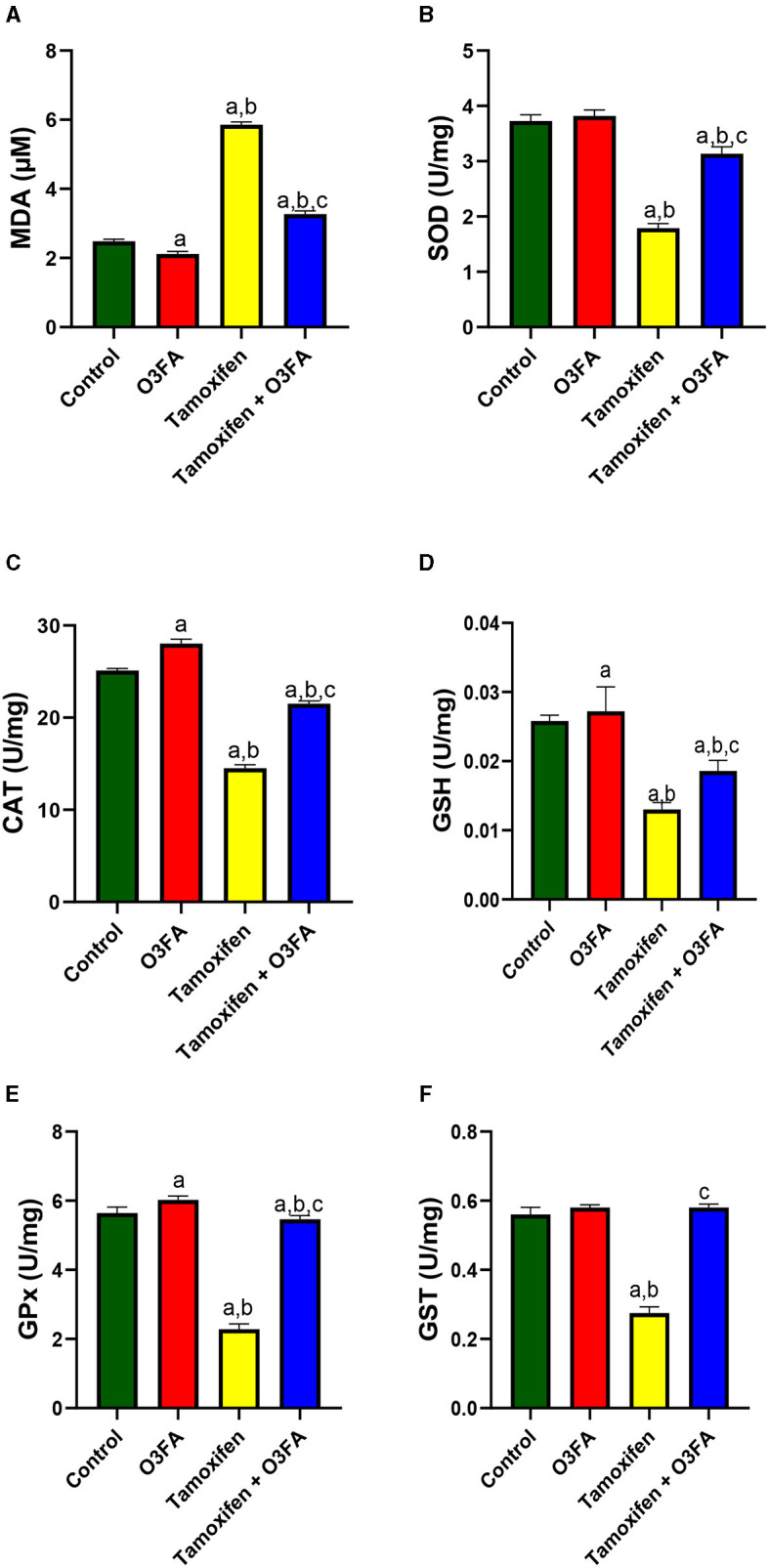
Effect of O3FA on testicular **(A)** malondialdehyde (MDA) **(B)** superoxide dismutase (SOD) **(C)** catalase (CAT) **(D)** glutathione (GSH) **(E)** glutathione peroxidase (GPx) **(F)** Glutathione-S-transferase (GST) in TAM exposed rats. ^a^*P* < 0.05 vs. control, ^b^*P* < 0.05 vs. O3FA; ^c^*P* < 0.05 vs. TAM. Data were analyzed by one way ANOVA and Tukey's *post-hoc* test. O3FA, Omega 3 fatty acids; TAM, Tamoxifen.

Also, Figure 7 showed that TAM administration led to a significant increase in testicular MDA and a decrease in testicular SOD, CAT, GSH, GPX, and GST. This observed redox imbalance was abrogated in animals that received O3FA and TAM co-treatment.

**Figure 8:**
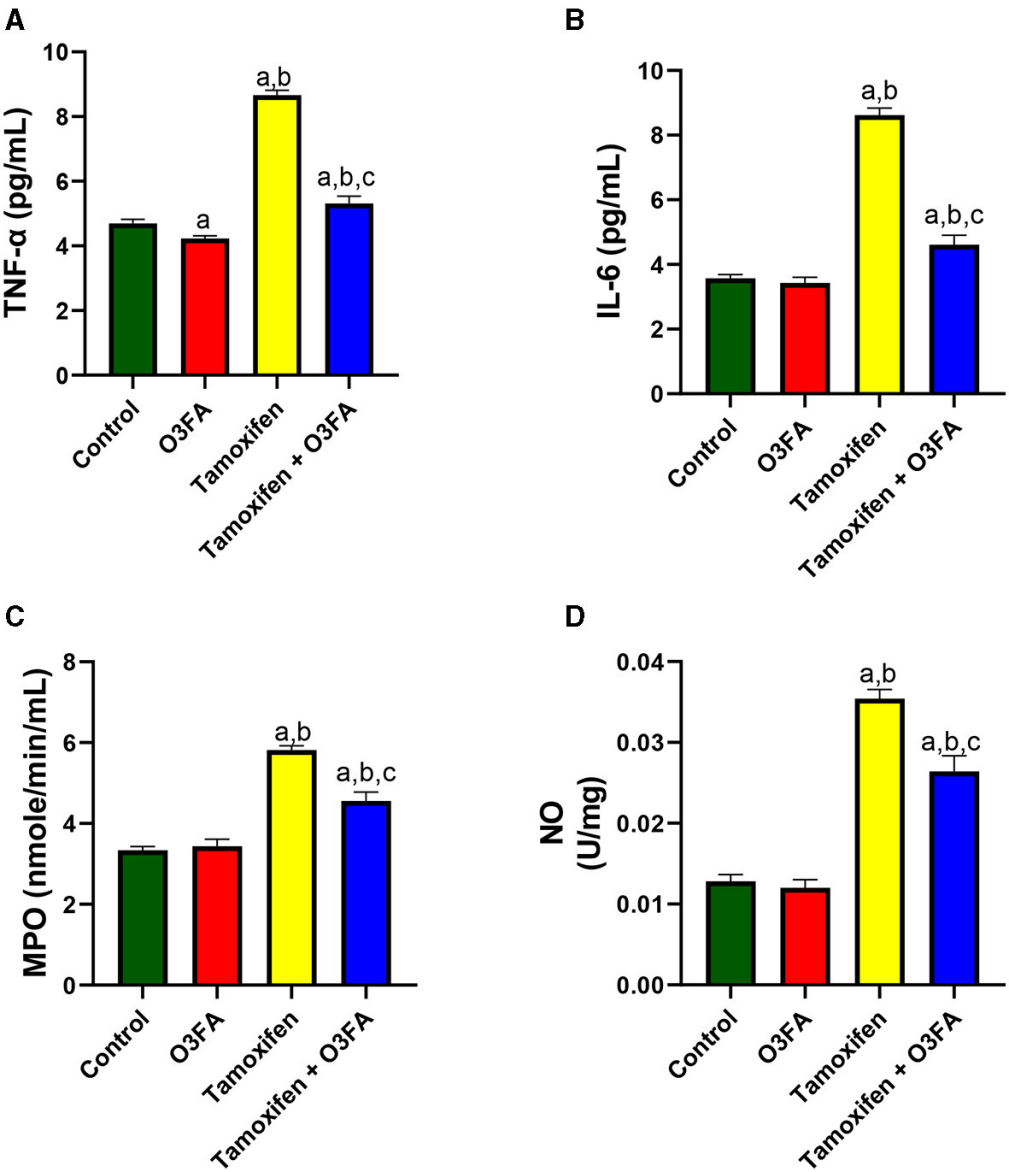
Effect of O3FA on testicular **(A)** tumor necrotic factor-alpha (TNF-α) **(B)** interleukin 6 (IL-6) **(C)** myeloperoxidase (MPO) **(D)** nitric oxide (NO) in TAM exposed rats. ^a^*P* < 0.05 vs. control, ^b^*P* < 0.05 vs. O3FA; ^c^*P* < 0.05 vs. TAM. Data were analyzed by one way ANOVA and Tukey's *post-hoc* test. O3FA, Omega 3 fatty acids; TAM, Tamoxifen.

Similarly, TAM treatment significantly led to an increase in testicular TNF-α, IL-6, MPO, and NO compared with the control groups (Figure 8). These observed TAM-induced inflammatory responses were ameliorated in animals treated with TAM and O3FA.

**Figure 9:**
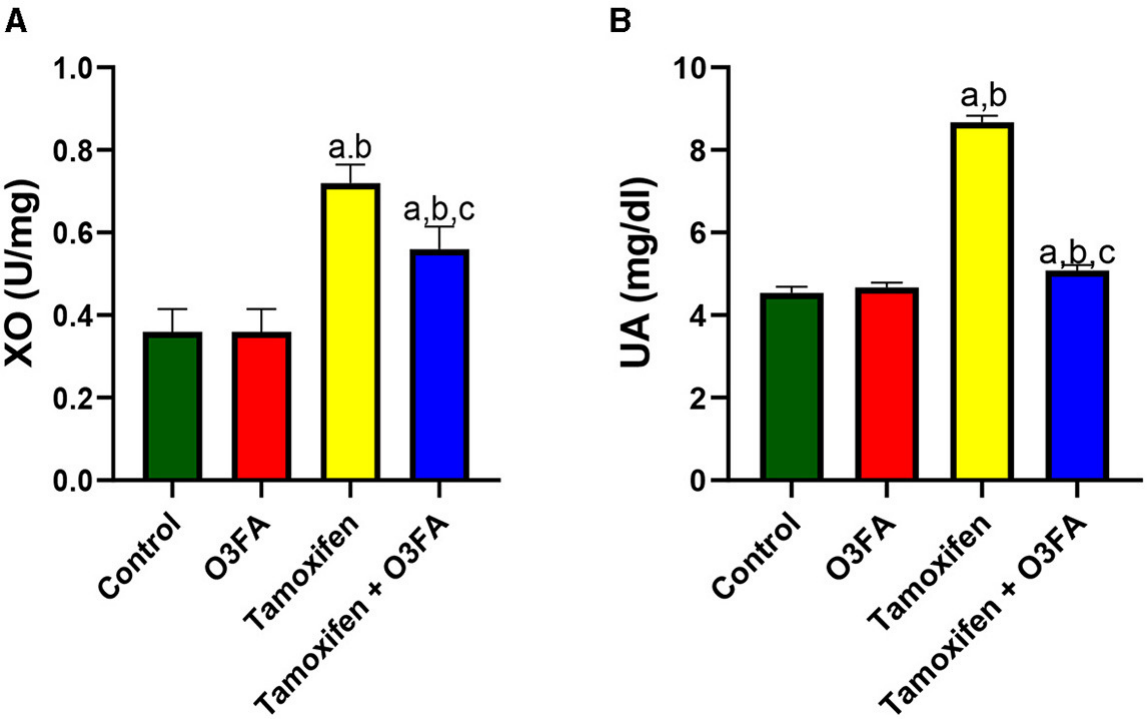
Effect of O3FA on testicular **(A)** Xanthine oxidase (XO) **(B)** uric acid (UA) in TAM exposed rats. ^a^*P* < 0.05 vs. control, ^b^*P* < 0.05 vs. O3FA; ^c^*P* < 0.05 vs. TAM. Data were analyzed by one way ANOVA and Tukey's *post-hoc* test. O3FA, Omega 3 fatty acids; TAM, Tamoxifen.

Furthermore, testicular XO and UA were significantly elevated in animals treated with TAM compared with the control (Figure 9). These observed TAM-induced XO/UA signaling distortions were blunted in animals treated with TAM and O3FA.

**Figure 10:**
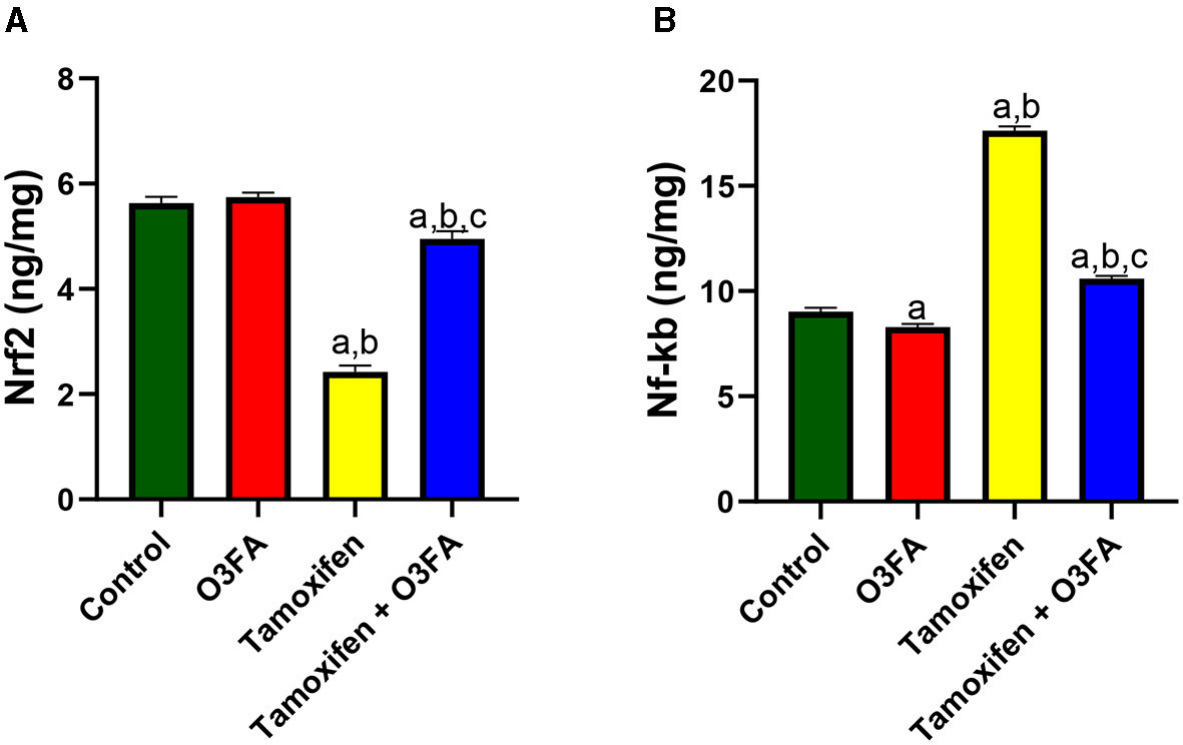
Effect of O3FA on testicular **(A)** Nrf2 **(B)** Nf-kb in TAM exposed rats. ^a^*P* < 0.05 vs. control, ^b^*P* < 0.05 vs. O3FA; ^c^*P* < 0.05 vs. TAM. Data were analyzed by one way ANOVA and Tukey's *post-hoc* test. O3FA, Omega 3 fatty acids; TAM, Tamoxifen.

Additionally, TAM administration led to a significant decrease in testicular Nrf2 and an increase in testicular Nf-κB compared with the control groups (Figure 10). This observed TAM-induced Nrf2/Nf-κB signaling distortion in TAM-treated animals was ameliorated in their counterparts treated with TAM and O3FA.

**Figure 11:**
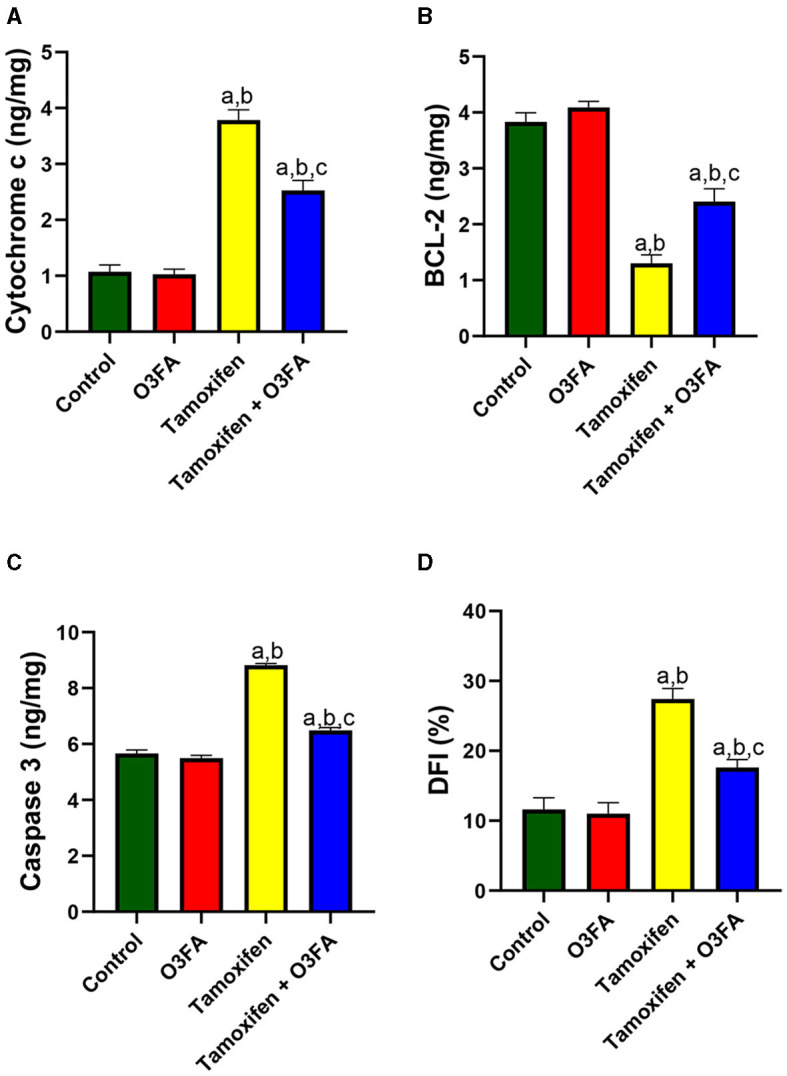
Effect of O3FA on testicular **(A)** cytochrome c **(B)** B-cell lymphoma 2 (BCl-2) **(C)** caspase 3 **(D)** DNA fragmentation index (DFI) in TAM exposed rats. ^a^*P* < 0.05 vs. control, ^b^*P* < 0.05 vs. O3FA; ^c^*P* < 0.05 vs. TAM. Data were analyzed by one way ANOVA and Tukey's *post-hoc* test. O3FA, Omega 3 fatty acids; TAM, Tamoxifen.

Finally, TAM exposure led to a significant increase in testicular cytochrome C, BCl-2, caspase 3, and DFI compared with the animals in the control group (Figure 11). TAM and O3FA co-treatment blunted this observed increase in apoptotic markers.

## Discussion

It has been sufficiently established that estrogen plays a major role in male reproductive system development and maintenance. In fact, the presence of ER α and β in the testicular (43, 44) and sperm cells (45, 46) indicates the cognate role of estrogen in testicular functions. Supportively, Korach (47) reported impaired testicular functions in ER α and β knockout mice. TAM is a potent nonsteroidal antiestrogen that has been recommended for managing breast cancer and gynecomastia. In fact, it has been recommended for treating idiopathic oligospermia despite insufficient data on its effectiveness (48, 49). However, these authors did not compare their findings with a placebo control, which is a fundamental aspect to consider when testing the real therapeutic effect of a drug. In fact, Rolf et al. (50) concluded that the beneficial role of TAM may not justify its side effects in healthy males after reviewing 29 clinical trials involving 1,586 patients. TAM has been reported to negatively impact male fertility status in different strains, including rats, monkeys, and dogs (1). Although different studies have studied the antiestrogenic and estrogenic effects of TAM, information on its effect on oxido-inflammatory response and apoptosis on testicular tissue is still lacking. Hence, we investigated the putative gonadotoxic effects of TAM and the possible role of redox imbalance, inflammation, and apoptotic response in TAM-induced testicular dysfunction. Also, we explored the possible ameliorative effect of O3FA on TAM-induced gonadotoxicity.

Our findings revealed that O3FA treatment inhibited the impaired sperm quality, steroidogenesis, HPG-axis, and surge in testicular injury markers following TAM exposure. These histological and biochemical events were accompanied by O3FA-induced amelioration of TAM-mediated distortion of Nrf2/Nf-κb signaling and the consequent redox balance, the suppression of inflammatory response, and cytochrome C-mediated apoptosis.

In this study, TAM administration led to a significant decrease in sperm motility, count, and normal morphology. This impaired sperm quality was accompanied by a significant decline in serum testosterone and steroidogenic enzymatic activities, which are in tandem with previous reports (13). Different mechanisms might be responsible for the observed spermatogenesis and steroidogenesis impairment. TAM might impair testicular function by disrupting the HPG axis activities or via direct testicular damage. In male reproduction, the hypothalamus is responsible for secreting GnRH, which stimulates LH and FSH secretion from the pituitary gland, which also stimulates the testis. The secreted LH stimulates steroidogenesis (testosterone and estrogen secretion), while FSH stimulates spermatogenesis. Additionally, testosterone and estrogen also play a dominant role in spermatogenesis. Testosterone and estrogens, in turn, inhibit the synthesis of the gonadotropins at the level of the pituitary or directly inhibit GnRH secretion from the hypothalamus (51). Thus, the disruption of the HPG axis activities at any level will directly impair testicular functions (spermatogenesis and steroidogenesis). The fact that TAM exposure led to a significant increase in gonadotropins (LH and FSH) might suggest that TAM-induced testicular dysfunction could be independent of the HPG axis activities rather than via direct testicular damage since circulatory LH was unable to stimulate the gonad (testis) to synthesize testosterone.

The fact that TAM exposure disrupted the normal testicular cytoarchitecture supports our claim that TAM might impair testicular function via direct testicular damage. Also, the increase in testicular injury markers (Lactate, LDH, GGT, and SDH) following TAM exposure further substantiates our claim. Additionally, spermatogenesis is a complex process that requires energy balance (52). Unfortunately, the observed increase in lactate is a marker of energy imbalance (53), which is an indication of impaired spermatogenesis and testicular degeneration (22). These findings corroborated previous findings of Marek et al. (54), who reported that TAM activities are associated with energy imbalance and an increase in lactate.

This direct testicular damage could result from oxidative stress or redox imbalance, which plays a key role in testicular functions (55, 56). Oxidative stress occurs when there is an imbalance between pro-oxidant generation and antioxidant activities. Oxidative stress, on the other hand, can stimulate different transcription factors to activate inflammatory pathways (57, 58). TAM treatment could impair testicular function via its oxido-inflammatory activities evidenced by an increase in testicular MDA, IL-6, TnF-a, MPO, and NO and a decrease in CAT, SOD, GSH, GST, and GPx. These observed TAM-induced oxido-inflammatory responses could be mediated by the increase in XO/UA signaling. An increase in XO and the consequent increase in UA has been implicated in lipid peroxidation (59). Although UA is an antioxidant, it becomes a pro-oxidant once produced in excess (60), thereby generating excessive ROS. Excessive ROS can overwhelm Nrf2 activities, the endogenous transcription factor responsible for maintaining redox balance (35). The consequent redox imbalance might activate Nf-κb, responsible for increasing pro-inflammatory gene induction, leading to an inflammatory response (61). The increase in Nf-κb will further inhibit Nrf2 activities, thereby leading to a further decline in the endogenous antioxidant activities. This observed XO/UA and Nrf2/Nf-κb-mediated oxido-inflammatory response following tamoxifen exposure agreed with the study of Ahmed et al. (62) and Schieber and Chandel (63), who reported that TAM can impair cellular functions via oxidative stress.

Additionally, excessive oxidative stress and inflammatory response can collaborate to stimulate apoptosis (64), which is another key factor that can be responsible for TAM-induced testicular dysfunction. The observed increase in testicular cytochrome c following TAM exposure could account for the observed TAM-induced apoptotic response. In mammals, the cytochrome c-initiated pathway is a key caspase activation pathway (65). Various apoptotic stimuli can stimulate the release of cytochrome c from the mitochondria, leading to a series of biochemical reactions that activate caspase and the consequent cell death. Mitochondria plays a major role in the redistribution of cytochrome c (66). Also, the anti-apoptotic protein (BCl-2) located predominantly at the outer mitochondria membrane assists in blocking Δψm reduction and cytochrome c release (67). Hence, during mitochondrial dysfunction, there is a leakage of cytochrome c from the mitochondria and a decrease in BCl-2 (66), thereby leading to caspase 3- 3-mediated apoptosis (68). Hence, it is plausible that the observed increase in testicular caspase 3 and DFI and decrease in testicular BCl-2 might be associated with the leakage of cytochrome c from the mitochondria of TAM-treated rats. Our guess that TAM disrupts testicular function via mitochondria dysfunction-mediated apoptosis corroborates the findings of Unten et al. (69) and Nazarewicz et al. (70).

Another key finding from this study is the therapeutic potential of O3FA against TAM-induced testicular dysfunction. This study revealed that O3FA ameliorated TAM-induced testicular dysfunction by decreasing testicular injury markers and oxido-inflammatory and apoptotic response, thus improving spermatogenesis, sperm quality, hormone synthesis, and testicular histoarchitecture. These findings agreed with previous studies that established the antioxidant (71), anti-inflammatory (22), and anti-apoptotic (72) effects of O3FA. Hence, it is safe to infer that the increase in testicular SOD, CAT, GSH, GPX, and GST and decrease in TNF-a, IL-6, MPO, and NO of TAM exposed rats showed that O3FA –driven repression UA release via XO activities downregulation probably modulated the Nrf2/Nf-κb signaling, thereby inhibiting the transcription of genes responsible for encoding pro-inflammatory cytokines and oxidative response. Furthermore, the observed increase in BCl-2 and decrease in caspase 3 and DFI in O3FA and TAM co-treated rats could also be associated with O3FA-associated decline in cytochrome c release from the mitochondria.

## Conclusion

The findings from this study revealed that O3FA ameliorated impaired sperm quality, hormonal imbalance, oxido-inflammatory response, and apoptosis by modulating XO/UA and Nrf2/NF-kb signaling and cytochrome c-mediated apoptosis in TAM-treated rats. These findings suggests the combination therapy with TAM and O3FA in the management of gynecomastia and breast cancer, since O3FA can help alleviate the side effects associated with TAM with respect to male fertility.

## Limitations

This study was conducted in healthy animals and we suggest a replica of it in gynecomastia subjects receiving TAM treatment. Additionally, downstream target genes responsible for maintain oxido-inflammatory response and apoptosis were not estimated using real-time PCR, western blot, immunohistochemistry, or TUNEL analysis (for apoptosis). However, the observed modulation of XO/UA and Nrf2/NF-kb and cytochrome c-mediated apoptosis accompanied by oxido-inflammatory response suggests the involvement of these pathways in TAM-induced testicular dysfunction.

## Data availability statement

The raw data supporting the conclusions of this article will be made available by the authors, without undue reservation.

## Ethics statement

The animal study was approved by the University of Ilorin Ethical Review Committee. The study was conducted in accordance with the local legislation and institutional requirements.

## Author contributions

AO: Conceptualization, Data curation, Formal analysis, Funding acquisition, Investigation, Methodology, Project administration, Resources, Software, Supervision, Validation, Visualization, Writing – original draft, Writing – review & editing. RA: Formal analysis, Investigation, Methodology, Project administration, Supervision, Validation, Visualization, Writing – review & editing. MH: Formal analysis, Funding acquisition, Investigation, Methodology, Project administration, Resources, Supervision, Validation, Visualization, Writing – review & editing. MB: Methodology, Project administration, Supervision, Validation, Visualization, Writing – review & editing. DO: Methodology, Project administration, Supervision, Visualization, Writing – review & editing. LO: Methodology, Project administration, Resources, Supervision, Validation, Visualization, Writing – review & editing.
